# Optimizing the Use of Chief Complaint & Diagnosis for Operational Decision Making: An EMR Case Study of the 2010 Haiti Earthquake

**DOI:** 10.1371/currents.dis.2c6c4d44dc0260af0867e0bc30b85aa7

**Published:** 2014-08-27

**Authors:** Alexandra T. Bambrick, Dina B. Passman, Rachel M. Torman, Alicia A. Livinski, Jennifer M. Olsen

**Affiliations:** The George Washington University, School of Public Health & Health Services, Washington, District of Columbia, USA; Department of Health and Human Services, Office of the Assistant Secretary for Preparedness and Response, Office of Emergency Management, Division of Fusion, Washington, District of Columbia, USA; Defense Threat Reduction Agency, Engility Corporation, Alexandria, Virginia, USA; National Institutes of Health, Office of Management, Office of Research Services, Division of Library Services, Bethesda, Maryland, USA; Department of Health and Human Services, Office of the Assistant Secretary for Preparedness and Response, Office of Emergency Management, Division of Fusion, Washington, District of Columbia, USA

## Abstract

Introduction: Data from an electronic medical record (EMR) system can provide valuable insight regarding health consequences in the aftermath of a disaster. In January of 2010, the U.S. Department of Health and Human Services (HHS) deployed medical personnel to Haiti in response to a crippling earthquake. An EMR system was used to record patient encounters in real-time and to provide data for decision support during response activities. Problem: During the Haiti response, HHS monitored the EMR system by recoding diagnoses into seven broad categories. At the conclusion of the response, it was evident that a new diagnosis categorization process was needed to provide a better description of the patient encounters that were seen in the field. After examining the EMRs, researchers determined nearly half of the medical records were missing diagnosis data. The objective of this study was to develop and test a new method of categorization for patient encounters to provide more detailed data for decision making. Methods: A single researcher verified or assigned a new diagnosis for 8,787 EMRs created during the Haiti response. This created a new variable, the Operational Code, which was based on available diagnosis data and chief complaint. Retrospectively, diagnoses recorded in the field and Operational Codes were categorized into eighteen categories based on the ICD-9-CM diagnostic system. Results: Creating an Operational Code variable led to a more robust data set and a clearer depiction emerged of the clinical presentations seen at six HHS clinics set up in the aftermath of Haiti’s earthquake. The number of records with an associated ICD-9 code increased 106% from 4,261 to 8,787. The most frequent Operational Code categories during the response were: General Symptoms, Signs, and Ill-Defined Conditions (34.2%), Injury and Poisoning (18.9%), Other (14.7%), Respiratory (4.8%), and Musculoskeletal and Connective Tissue (4.8%). Conclusion: The Operational Code methodology provided more detailed data about patient encounters. This methodology could be used in future deployments to improve situational awareness and decision-making capabilities during emergency response operations.

## Introduction

The practice of disaster medicine is evolving with the introduction and use of electronic medical records (EMRs) and electronic documentation in field operations.^1^ The EMR serves as a real-time patient encounter health record that allows clinical staff to enter, store, and share data with colleagues across time and space without the use of paper. This technology is capable of streamlining clinician workflow, improving data collection, improving quality of care, and increasing efficiency.^2^ The US Department of Health and Human Services (HHS), Office of the Assistant Secretary for Preparedness and Response (ASPR), National Disaster Medical System (NDMS) first deployed its EMR system in support of the HHS responses to Hurricanes Gustav and Ike in 2008. Since then, EMRs have been incorporated into most disaster responses, as NDMS personnel have been trained to use the EMR for patient documentation.

In January and February 2010, HHS deployed 1,100 personnel in support of the crippling Haiti earthquake that struck on January 12^th^.^3^ Patients were triaged and treated at HHS clinical sites established throughout the impact zone. For many of these encounters, EMRs were captured from January 18 to February 24, 2010.

Data were entered into the EMR via handheld mobile devices and laptops during registration, triage, treatment, and discharge. The EMR automatically assigned each patient encounter record with a unique identifier as well as a registration date and time stamp. The system allowed clinicians to enter free text for chief complaints and choose between selecting preloaded codes from the *International Classification of Diseases, Ninth Revision, Clinical Modification (ICD-9-CM)* 4 or entering free text for discharge diagnoses.

The staff within ASPR’s Office of Emergency Management (OEM) is responsible for information and data analysis for decision support for ASPR’s preparedness and response activities. During the Haiti response, OEM staff rapidly recoded patient encounters into seven Rapid Diagnostic groups (Acute, Chronic, Mental Health, Injuries, OB/GYN, Routine, and No Data) for situational awareness and decision-making. The majority of these encounters were of an acute nature (53%), while injuries only accounted for 22% of the patient encounters (Figure 1).

**Patient encounters by rapid diagnostic groups:**
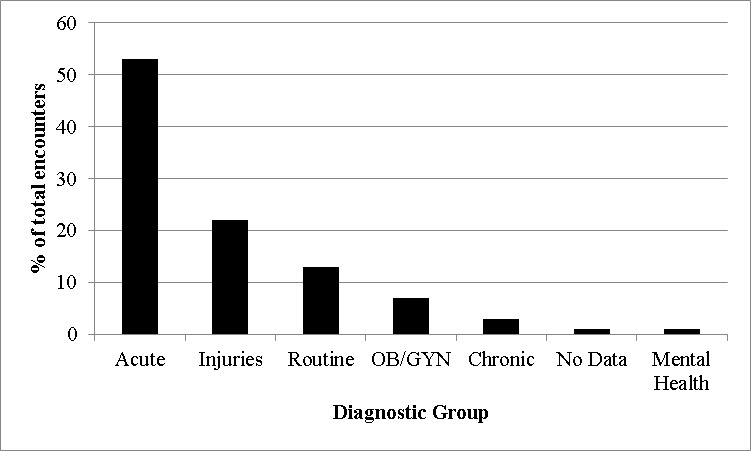

The rapid categorization methodology used to code the patient encounters into seven broad Rapid Diagnostic Groups was initially based on discharge diagnosis alone, and later included chief complaint information. However, there was no standardized process for performing this recoding. As such, the process changed incrementally over time as staff became more familiar with the response EMR data and became more experienced with the recoding. This recoding was initially done for two reasons. First, ASPR leadership and analytical staff had pre-agreed upon the need to roll data up into a limited number of categories to improve high-level surveillance and understanding of the response. Second, as the response progressed, it was determined that the response personnel did not sufficiently code discharge diagnosis for many of the patient encounters, which resulted in thousands of records without a discharge diagnosis and hundredsof records coded with a diagnosis that had no relevance to the chief complaint. These reasons necessitated the need for a more detailed record review in order to categorize encounters. The rapid categorization methodology was sufficient for providing a high-level overview of the data during the response; however, it was not robust enough to support more in-depth inquiry and a detailed understanding of the response and the conditions for which people were treated at HHS sites. Therefore, the objective of this study was to develop and test a new method of categorization for patient encounters to provide more detailed data for decision making. A novel approach was developed and used retrospectively to evaluate discharge diagnosis and chief complaint EMR data together to determine if more valuable insight is available than from either variable independently.

## Methods

A literature review of best practices for categorizing patient encounter records during a disaster was conducted in MEDLINE/PubMed using variations of the following terms: “emergency response”, “disaster response”, “disaster relief”, Haiti, “diagnosis and chief complaint”, “patient categorization”, “field categorization”, “syndromic surveillance”, “disaster surveillance”, “disaster patient surveillance system” and “best practice”. No strong evidence to support categorizing or grouping patient records during a disaster response by any specific means was identified. The most relevant recommendations were from *MMWR*s issued by the Centers for Disease Control and Prevention (CDC) after disaster responses for Hurricanes Katrina and Rita in 2005.^5 ^^,^ 6 ^,^ 7 In the *MMWR*s, the CDC reported their collection of data using a specific coding system and reported those data organized by either systems or syndromes on a large scale.^5 ^^,^ 6 ^,^ 7

Retrospectively, a single researcher (AB) with a clinical background in acute care nursing assigned the Haiti patient encounters the closest ICD-9-CM codes (i.e., Operational Code) to allow the grouping of patient encounter records into categories that more completely described the encounters and, as a result, the response.^4^ ICD-9-CM coding was selected, as it was the standard diagnostic system used in the Haiti response, as well as in previous HHS deployments. For each record, clinicians had the ability to document multiple diagnoses distinguished as “diagnosis text,” “discharge dx 1,” and “discharge dx 2” in the data set. The researcher used “discharge dx 1” (i.e., primary discharge diagnosis) for this study as it is standard practice that a clinician would document the primary and most severe diagnosis in this field. A chief complaint was valid when the chief complaint field contained patient signs and symptoms. If multiple chief complaints were recorded with no diagnosis, the primary chief complaint was selected based on severity, using the researcher’s clinical knowledge of triage based on the Emergency Nurses Association standards.^8^

The initial methodology to assign an Operational Code was simple (Figure 2, white scale and solid arrows). If an ICD-9-CM diagnosis was present in the “discharge dx 1” field—meaning it was assigned in the field during the response—the Operational Code assigned was the exact same. If there was no primary discharge diagnosis recorded, the recorded primary chief complaint was used to retrospectively assign the Operational Code. If no chief complaint or any discharge diagnosis was recorded, the patient encounter record was marked for removal from the final data set.

Early in the process of reviewing records; however, a preponderance of the use of the Herpes Zoster ICD-9-CM code was identified. Within the EMRs the code 053.79 was displayed as “Other” and subsequently 855 records (9.4%) had this code recorded as the primary diagnosis. However, this code is actually a diagnosis for “Herpes Zoster, with other specified complications, other.” As herpes zoster is predominately seen in individuals older than 65, and even then has an incidence of only 3.9 to 11.8 cases per 1,000 (0.39%to 1.18%),^9^ the researchers questioned the validity of diagnosing 855 individual encounters of herpes zoster in the aftermath of the earthquake in a developing country with a population skewed towards those younger than 65 years of age. Further examination of these records revealed that the chief complaints for these patient encounters included fractured bones, fatigue, nausea and vomiting, and more—not complaints specifically associated with herpes zoster. Therefore, the initial methodology was deemed insufficient due to the mismatch of the chief complaint and diagnosis and was suspended. The Operational Code methodology was then developed and introduced.

In the Operational Code methodology, a step to confirm that the recorded chief complaint and primary discharge diagnosis matched was incorporated in order to account for possible clinician data entry challenges and to ensure better data fidelity, (Figure 2, gray scale decisions with dashed arrows). The researcher started recoding again and individually examined each patient encounter record. If a recorded primary discharge diagnosis was entered, the recorded chief complaint was reviewed if present. If no chief complaint was entered, the Operational Code was assigned the value of the “discharge dx 1” (e.g., nothing was changed) by the researcher. When a chief complaint was present, it was determined if it corresponded to the primary discharge diagnosis. If these corresponded, the Operational Code assigned was again the value of the “discharge dx 1” (e.g., nothing was changed). If the chief complaint and primary discharge diagnosis did not correspond, an Operational Code was assigned using the primary chief complaint because it was assumed the clinician in the field made a coding error or that triage was inaccurate.

Nearly half of the EMRs did not have a diagnosis recorded; in these cases the primary chief complaint was the only option in assigning the Operational Code without a full chart review, but even then data quality varied. The primary chief complaints, which were recorded as free text, varied greatly. For example, there were 77 records where “flu,” “flu like symptoms,” or “influenza like illness (ILI)” were listed as the chief complaint with no diagnosis; however, according to ICD-9-CM diagnostic coding, chief complaints of “flu like symptoms” are given a specific diagnosis (code) after tests have been performed, that were not available during the Haiti response. ILI actually refers to 29 specific ICD-9-CM codes. This situation created a problem because the researcher did not want to assume that all of these encounters were truly influenza, nor did the researcher want to generalize the data to the point it would not be useful. Therefore, all chief complaints of this nature were assigned the code 780.99 for “General symptoms/Other general symptoms/Other.” No other chief complaint used this code, so in analyzing the data, the team could account for this subset of patient encounters specifically if need be.

**Initial and final operational code methodology:**
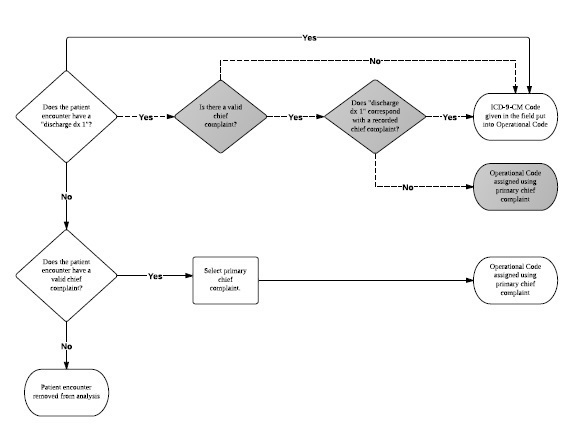
White scale and solid arrows: Initial operational code methodology. Gray scale and dashed arrows: Decisions added to the initial methodology to formulate the final operational code methodology.

Patient encounter records were pulled from the NDMS Health Information Repository if the associated deployment name was equal to “Haiti.” For comparison of the field-assigned primary discharge diagnoses and the researcher-assigned Operational Codes, each was assigned a diagnostic category (i.e. Discharge Diagnosis Category and Operational Code Category). Records were recoded into eighteen mutually exclusive diagnostic categories. Seventeen were based on the ICD-9-CM coding classification system, which rolls individual diagnosis codes into groups by system. For comparison purposes, an eighteenth category was created and named “Other” and consisted of primary discharge diagnosis values of ICD-9-CM V-codes and E-codes. Those records without a field-assigned primary discharge diagnosis were not assigned a diagnosis category. Category names were shortened from the traditional ICD-9-CM standard for ease of use. Original category names can be found in Appendix 1.

Records were removed from the data set if: no primary discharge diagnosis and no chief complaint were entered; the record was marked as a duplicate by staff in the field; or the record corresponded to the treatment of response personnel. Records documenting treatment of response personnel were removed from the data set to reduce information bias that could be introduced by including the records of people who were not directly impacted by the earthquake. Personnel records were identified in one of two ways. The first method was to exclude those records that had been coded by the staff entering data into EMR as belonging to a federal responder. The second method was to flag those records as being those of personnel that included information in the chief complaint such as “responder” or “needle stick”.

The relative frequencies of diagnoses in each of the eighteen mutually exclusive categories were determined for both Discharge Diagnosis category (field-assigned) and Operational Code category (retrospectively researcher-assigned) diagnoses/codes.

A chi-square test for equal proportions was used to determine whether the distribution of the patient encounter records within each diagnostic category were significantly different before and after applying our Operational Code methodology (Figure 2). Records that were not assigned an ICD-9-CM code in the field were excluded from this analysis.

To assess the agreement in diagnosis categorization between the field-assigned discharge diagnosis and the researcher-assigned Operational Code, Cohen’s Kappa statistics were runfor all records that had a field-assigned discharge diagnosis.^10^ The same analysis was also run stratifying by diagnosis groupings.

To test intra-rater reliability through Cohen’s Kappa coefficients, a weighted, random sample (n=454) was derived from the records that already had a diagnosis (n=4,261). This random sample represented approximately 10% of those records that were not assigned a discharge diagnosis in the field and were assigned an Operational Code using the primary chief complaint. The ICD-9-CM-based Operational Code categories were the stratification variable. Once the sample was obtained the same researcher (AB) used the established Operational Code methodology (Figure 2) to blindly assign each record an Operational Code.

Cohen’s Kappa coefficients were calculated to test for intra-rater reliability by comparing the diagnosis categories from the researcher’s (AB) original and repeat assessments. After taking into account the amount of agreement that would take place by chance alone, the magnitude of the Kappa coefficient represents the extent of agreement after a single researcher recodes a sample two or more times.^11^

In this study, the statistical significance level was p <0.05. Based on the interpretation of Kappa statistics by Viera and Garrett,^12^ Kappa values were interpreted as being in “slight agreement” (0.01-0.20), “fair agreement” (0.21-0.40), “moderate agreement” (0.41-0.60), “substantial agreement” (0.61-0.80), “almost perfect agreement” (0.81-0.99), and “perfect agreement” (1.0). SAS Enterprise Guide Version 5.1 (SAS Institute Inc., Cary, NC) was used for all analysis.

This research did not meet requirements for Department of Health and Human Services IRB review and was deemed exempt.

## Results

For this analysis, 296 records (3%) were removed from the raw EMR data. The researchers excluded 107 records that were identified as belonging to HHS staff, 181 records that were deemed incomplete and 8 records that were recorded as duplicates. The final data set consisted of 8,787 patient encounter records (Figure 3).

**Patient encounter record inclusion and exclusion criteria:**
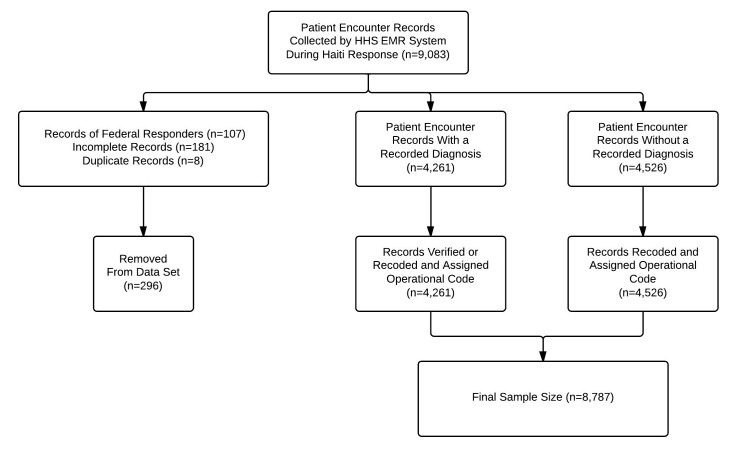

The majority of encounters were with females (51.8%). The mean age of patient encounters was 25.86 (SD 19.34). Most of the patient encounters were coded as non-urgent (73.2%) at triage. Emergent encounters accounted for only 3.2% of patient encounters.

As shown below in Table 1, the top five ICD-9-CM diagnoses assigned in the field were those of Parasitic Diseases, Injury, Other, General, and Respiratory. Parasitic diseases accounted for 26% of the total data set with Injury accounting for 22% of the cases assigned a code in the field. Yet, if adjusted to include those records missing an ICD-9-CM code, Parasitic Disease accounted for 13% and Injury just 11% of all cases. The large percentage of diagnoses falling into the Other category is due to a large number of records pertaining to follow-up patient encounters that were coded using V-codes. These included, but were not limited to, patients who needed bandage checks and changes or patients who needed to be re-evaluated after a procedure.

**Table 1: Frequency of discharge diagnosis codes by category from HHS Haiti response (n=4,261):** 

Diagnostic Category (ICD-9-CM Codes) (n=4,261)	n (%)
Infections and Parasitic Diseases (001-139)	1117 (26.2)
Injury and Poisoning (800-999)	926 (21.7)
Other (V-Codes, E-Codes)	689 (16.1)
General Symptoms, Signs, and Ill-Defined Conditions (780-799)	610 (14.3)
Respiratory (460-519)	191 (4.5)
Digestive (520-579)	152 (3.6)
Genitourinary (580-629)	126 (3)
Endocrine, Nutritional, Metabolic, Immunity (240-279)	90 (2.1)
Skin, Subcutaneous (680-709)	76 (1.8)
Nervous and Sense Organs (320-389)	70 (1.6)
Musculoskeletal and Connective Tissue (710-739)	55 (1.3)
Pregnancy, Childbirth, Puerperium (630-679)	45 (1.1)
Mental Disorders (290-319)	35 (0.8)
Circulatory (390-459)	29 (0.7)
Perinatal (760-779)	24 (0.6)
Neoplasms (140-239)	18 (0.4)
Congenital Anomalies (740-759)	6 (0.1)
Blood and Blood-Forming Organs (280-289)	2 (0.1)

Table 2 shows the frequencies of recoded encounter records using the same diagnostic categories. When using the Operational Code Category as the analysis variable the top five categories were General Symptoms (34.2%), Injury and Poisoning (18.9%), Other (14.7%), Respiratory Diseases (4.8%), and Musculoskeletal (4.8%).

**Table 2: Frequency of operational codes by category from HHS Haiti response (n=8,787):** 

Diagnostic Category (ICD-9 CM Codes) (n=8787)	n (%)
General Symptoms, Signs, and Ill-Defined Conditions (780-799)	3003 (34.2)
Injury and Poisoning (800-999)	1660 (18.9)
Other (V-Codes, E-Codes)	1293 (14.7)
Respiratory (460-519)	424 (4.8)
Musculoskeletal and Connective Tissue (710-739)	421 (4.8)
Genitourinary (580-629)	364 (4.1)
Nervous and Sense Organs (320-389)	347 (4)
Infections and Parasitic Diseases (001-139)	326 (3.7)
Digestive (520-579)	264 (3)
Endocrine, Nutritional, Metabolic, Immunity (240-279)	154 (1.8)
Skin, Subcutaneous (680-709)	152 (1.7)
Pregnancy, Childbirth, Puerperium (630-679)	122 (1.4)
Circulatory (390-459)	117 (1.3)
Mental Disorders (290-319)	78 (0.9)
Blood and Blood-Forming Organs (280-289)	19 (0.2)
Neoplasms (140-239)	18 (0.2)
Perinatal (760-779)	18 (0.2)
Congenital Anomalies (740-759)	7 (0.1)

The chi-square test for equal proportions demonstrated that the categorization methodology led to a different distribution of medical records within each diagnostic category when comparing Discharge Diagnosis Category (n=4,261) and Operation Code Category (n=8,787) (Table 3). Fifteen of the categories had a significantly different number of records after application of the categorization methodology (p<0.05). Three categories had no statistically significant change in the number of records after categorization: Neoplasms (p=1.0000), Congenital Anomalies (p=0.7815), and Perinatal (p=0.3545).

A second chi-square test for equal proportions demonstrated that the Operational Code methodology led to a different distribution of medical records within each diagnostic category when comparing those records that were assigned a discharge diagnosis in the field (n=4,261) versus those that were not (n=4,526) (Table 4). Ten of the categories had a significantly different number of records (p<0.05). Eight categories had no statistically significant change in the number of records: Blood and Blood-Forming Organs (p=0.8185), Mental Disorders (p=0.0700), Circulatory (p=0.7815), Genitourinary (p=0.2489), Skin (p=0.0516), Congenital Anomalies (p=0.2568), Perinatal (p=0.3558), and Injury and Poisoning (p=0.1281).

**Table 3: Chi square results:** 

	Discharge Diagnosis Category vs. Operational Code Category		Assigned Discharge Dx vs. Not Assigned Discharge Dx	
Diagnostic Code Category	χ2	P value	χ2	P value
Infections and Parasitic Diseases (001-139)	433.60	**< 0.0001**	113.08	**< 0.0001**
Neoplasms (140-239)	0.00	1.0000	8.00	**0.0047**
Endocrine, Nutritional, Metabolic, Immunity (240-279)	16.79	**< 0.0001**	28.29	**< 0.0001**
Blood and Blood-Forming Organs (280-289)	13.76	**0.0002**	0.05	0.8185
Mental Disorders (290-319)	16.36	**< 0.0001**	3.28	0.0700
Nervous and Sense Organs (320-389)	184.00	**< 0.0001**	21.81	**< 0.0001**
Circulatory (390-459)	53.04	**< 0.0001**	0.08	0.7815
Respiratory (460-519)	88.27	**< 0.0001**	14.35	**0.0002**
Digestive (520-579)	30.15	**< 0.0001**	24.24	**< 0.0001**
Genitourinary (580-629)	115.60	**< 0.0001**	1.33	0.2489
Pregnancy, Childbirth, Puerperium (630-679)	35.50	**< 0.0001**	27.57	**< 0.0001**
Skin, Subcutaneous (680-709)	25.33	**< 0.0001**	3.79	0.0516
Musculoskeletal and Connective Tissue (710-739)	281.42	**< 0.0001**	76.11	**< 0.0001**
Congenital Anomalies (740-759)	-	-	1.29	0.2568
Perinatal (760-779)	0.86	0.3545	0.89	0.3458
General Symptoms, Signs, and Ill-Defined Conditions (780-799)	1584.96	**< 0.0001**	252.63	**< 0.0001**
Injury and Poisoning (800-999)	208.34	**< 0.0001**	2.32	0.1281
Other (V-Codes, E-Codes)	184.06	**< 0.0001**	136.87	**< 0.0001**

The overall agreement between Discharge Diagnosis category and Operational Code category was moderate (к=.54; 95% CI=.52 to .55) (Table 4).[12] There was variability in agreement across the diagnosis categories. Agreement between the diagnostic category and operational code was perfect for diseases of the digestive system (к=1.00; 95% CI=1.00 to 1.00), and almost perfect for diseases of the respiratory system (к=.84; 95% CI=.81 to .88), of the endocrine system (к=.89; 95% CI=.84 to .93), and of the skin and subcutaneous tissue (к=.85; 95% CI=.79 to .91). The agreement was lowest for the Other category (к=.17; 95% CI=.14 to .21). Intra-rater reliability results in Table 4 shows un-weighted Kappa values for intra-rater reliability ranged from 0.38 to 1.00. Two of the fifteen diagnostic categories analyzed had perfect agreement, while six had substantial agreement. The overall Kappa value was 0.79 (95% CI 0.75-0.83), demonstrating that the patient encounter recoding methodology was repeatable by the researcher.

**Table 4: Kappa Statistics for category comparisons and inter-rater reliability:** 

	Discharge Diagnosis Category vs. Operational Code Category (n=4,261)		Intrarater Reliability (n=454)	
ICD-9-CM Diagnostic Category	К (95% CI)	Agreement Level	К (95% CI)	Agreement Level
Infections and Parasitic Diseases (001-139)	0.30 (0.27, 0.33)	Fair	0.85 (0.66, 1.00)	Almost perfect
Injury and Poisoning (800-999)	0.49 (0.46, 0.52)	Moderate	0.73 (0.66, 0.80)	Substantial
Other (V-Codes, E-Codes)	0.21 (0.18, 0.25)	Fair	0.38 (0.22, 0.53)	Fair
General Symptoms, Signs, and Ill-Defined Conditions (780-799)	0.62 (0.60, 0.65)	Substantial	0.90 (0.86, 0.94)	Almost perfect
Respiratory (460-519)	0.84 (0.81, 0.88)	Almost perfect	0.91 (0.81, 1.00)	Almost perfect
Digestive (520-579)	1.00 (1.00, 1.00)	Perfect	1.00 (1.00, 1.00)	Perfect
Genitourinary (580-629)	0.77 (0.71, 0.82)	Substantial	0.81 (0.68, 0.95)	Almost perfect
Endocrine, Nutritional, Metabolic, Immunity (240-279)	0.89 (0.84, 0.93)	Almost perfect	0.89 (0.67, 1.00)	Almost perfect
Skin, Subcutaneous (680-709)	0.85 (0.79, 0.91)	Almost perfect	0.83 (0.60, 1.00)	Almost perfect
Nervous and Sense Organs (320-389)	0.61 (0.53, 0.69)	Substantial	0.76 (0.62, 0.90)	Substantial
Musculoskeletal and Connective Tissue (710-739)	0.51 (0.42, 0.60)	Moderate	0.75 (0.61, 0.88)	Substantial
Pregnancy, Childbirth, Puerperium (630-679)	0.62 (0.52, 0.71)	Substantial	-	-
Mental Disorders (290-319)	0.70 (0.59, 0.82)	Substantial	0.66 (0.23, 1.00)	Substantial
Circulatory (390-459)	0.60 (0.48, 0.72)	Moderate	0.80 (0.52, 1.00)	Substantial
Perinatal (760-779)	0.34 (0.14, 0.54)	Fair	1.00 (1.00, 1.00)	Perfect
Neoplasms (140-239)	0.54 (0.34, 0.75)	Moderate	-	-
Congenital Anomalies (740-759)	0.73 (0.43, 1.00)	Substantial	-	-
Blood and Blood-Forming Organs (280-289)	0.33 (-0.01, 0.68)	Fair	-	-
Overall Kappa	0.51 (0.49, 0.53)	Moderate	0.79 (0.75, 0.83)	Substantial
*Overall Kappa for Intrarater reliability was adjusted to exclude categories not in sample				

## Discussion

The Operational Code methodology more than doubled the number of encounter records coded using the ICD-9-CM data standard (8,787 vs. 4,261). It also increased the granularity of the Rapid Diagnostic Group approach by increasing the number of categories for analysis from 7 to 18. The Rapid Diagnostic categories and the Operational Code categories are not readily comparable, but a few general differences between them can be observed. First, the original methodology used 7 categories, including “No Data”, that coded encounter records into mutually-exclusive groups based on a general mixed schema that identified encounters as being of a certain duration or requiring a certain level of care (“Acute”, “Chronic, or “Routine”) or being of a traumatic nature (“Injury”) or of a specific type (“OB/GYN” or “Mental”). By mixing types of visits in the coding, yet only allowing for one category per encounter record, acute obstetric encounters, for example, were being characterized as “OB/GYN” rather than “Acute”, skewing the high-level picture. The advanced methodology leveraged the ICD-9-CM system that consistently categorizes diagnosis codes by body system allowing for a more consistent understanding of the data, enabling a more robust analysis of the data from the Haiti response.

Using the ICD-9-CM categories to describe the patient encounters prior to using the Operational Code methodology provided limited value as over 50% of the records did not contain a primary discharge diagnosis and could not be readily categorized. By assigning an Operational Code to all of the records and then putting them into categories the full granularity of the data is revealed. Before implementing the Operational Code methodology, Diseases of the Musculoskeletal System and Connective Tissue accounted for only 1.3% (n=55) of patient encounters and ranked 11th, while after implementing the methodology they accounted for 4.8% (n=4210) of patient encounters and ranked 5th. Conversely, Infections and Parasitic Diseases accounted for 26.2% (n=1,117) of patient encounters prior to assigning an Operational Code and ranked 1st, while they accounted for only 3.7% (n=326) of patient encounters after recoding and ranked 8th. The revised burden of Infections and Parasitic Diseases, 22.5% lower than initial estimates, provides decision makers with a more accurate insight into the nature of the Haiti earthquake response that can be used for future planning and operations. Alternatively, while Injuries and Poisonings accounted for 21.7% (n=926) and 18.9% (n=1,660) of encounters (respectively) and remained ranked as the 2nd highest category both before and after implementing the recoding methodology, the burden increased by 734 encounters. This difference can more realistically inform response leadership as they estimate the personnel and supplies needed to treat such a large number of patients with these related diagnoses.

Additional analysis showed that large proportion of records that were missing a field-assigned discharge diagnosis fell into the General Symptoms, Signs, and Ill-Defined Conditions category (42.8%) after being recoded, which is higher than the final burden of the same category after applying the methodology (34.1%). Among the same subset, Nervous and Sense Organs made the top five (4.8%) after being recoded, where for records with a field-assigned discharge diagnosis it did not (1.6%).

Statistical analysis of the data provided other insights about the impact of the Operational Code methodology on the distribution of records among the 18 categories. Chi-square tests of homogeneity that compared the data before and after implementing the methodology (i.e., Discharge Diagnosis category vs. Operational Code category) resulted in all but 2 of the categories having a significant difference. Those 2 categories (Neoplasms and Perinatal), however, only accounted for 0.4% (n=36) of all encounter records after recoding. The Congenital Anomalies category was removed from this analysis due to too few records falling into this category. Comparison of categories of records that were and were not assigned discharge diagnoses in the field also had many significant findings. Of the 18 categories, 10 had statistically significant changes in distribution after recoding. Again, those that did not (Blood and Blood-Forming Organs, Mental Disorders, Circulatory, Genitourinary, Skin and Subcutaneous, Congenital Anomalies, Perinatal, and Injury and Poisoning) accounted for 26.4% (n=1,196) of encounters. These tests confirmed our hypothesis that applying the methodology would impact the distribution of records across the categories.

Our Kappa statistic findings comparing the agreement within categories for Discharge Diagnosis and Operational Code are consistent with the findings of Fleischauer^13^ and Begier^14^ despite their focus on syndromes while our work was focused on diagnostic categories. When looking at domestic emergency departments, Fleischauer^13^ and Begier^14^ found that there is high variability in agreement values across various syndrome classifications. However, our high level of agreement for both respiratory and digestive system diseases aligned with the findings of Begier^14^ who identified chief complaint and discharge data aligned for that system. The low level of agreement between the Other category from the Discharge Diagnosis category and Other from the Operational Code category aligned with our hypothesis that additional effort on coding encounters provided more granularity, as compared to the use of primary discharge diagnosis alone.^13^ The perfect agreement existing in the diseases of the digestive system may have been due to low number of encounters in this category before recoding (n=152).

The variability in agreement between Discharge Diagnosis category and Operational Code category may have been an outcome due to variation across ages or genders, as younger patients are known to have a higher discordance between chief complaints and discharge diagnoses.^13^ It is also unclear if the variations identified were the result of encounters with chief complaints that could have been associated with multiple discharge diagnoses.^13^

In this study, the patient encounter recoding methodology was deemed highly repeatable by the single researcher (AB), as the Kappa value ranged from 0.38 to 1.00 and the overall Kappa value was 0.79.The high level of agreement may be due to the operational code methodology, which aimed to minimize bias during recoding. Additionally, the researcher was blinded to the first Operational Code that was assigned and a random sample of the final data set was provided to the researcher.

At the very least, the EMR system, while not ideally designed to capture robust data during the Haiti response, did collect a multitude of information that retrospectively has proven informative regarding the types of patient encounters actually seen by HHS personnel. In addition, HHS learned how their EMR system worked well and where improvements were needed. The capturing of so much data is a vast improvement over paper records and allowed, albeit difficult, a detailed review of the information retrospectively. With further enhancements and additional training of personnel on the use of the EMR and the importance of data entry, the EMR has the strong potential to better support robust surveillance in the future.

## Limitations

While working in a stressful, austere environment at times, NDMS personnel did not record information within all of the fields in the EMR system. A discharge diagnosis was missing for nearly half of the records and our methodology discovered that for many of the records, the discharge diagnosis was possibly improperly coded. Additionally, there may have been records which were duplicates, but were not marked as so in the system. This could lead to a non-differential classification bias where some diagnoses corresponding to one patient may have been counted repeatedly. Lastly, there is a possible bias limitation due to translation. The national language of Haiti is Haitian Creole and many residents do not speak English or French. In this response, personnel who spoke Haitian Creole, as well as friends and family members, served in the role of translators. Professional medical translators were not used or widely available, which likely impacted both the data (especially for patient-provided fields like chief complaint) as well as the clinical care.^15 ^^,^ 16 ^,^ 17

The use of just one researcher and the retrospective design exposed the study to bias. However, the researcher took care not to make assumptions when assigning codes to avoid classification bias. The retrospective nature of this analysis prohibited complete accuracy due to the inability to verify data with providers in the field during the response.^18 ^^,^ 19 ^,^ 20

The Operational Code methodology does not account for acuity or duration of symptoms. This may present limitations to a decision maker’s ability to make staffing and medical asset decision based on these categories alone. Therefore this methodology should be combined with other data elements, such as incoming acuity, to understand the full scope of the patient population and its needs.

Sensitivity, specificity, and predictive value analysis could not be done with this data because the diagnosis may not have been mutually exclusive in this system, and there was not a confirmed standard for data entry at any of the sites.^21^

A limitation to utilizing the Kappa statistic is its dependence on the prevalence of the condition being detected,^22^ which may have been what we are seeing amongst the rarer conditions in this data set. Conditions with higher clinical significance may have been more likely to have a diagnosis recorded in the field. The authors also did not look at potential variation in agreement levels between sites within each given category, which may have been a potential root of the results.^14^

In this study, the single researcher waited over a year before recoding a subset of the data to support the analysis of intra-rater reliability. Sim and Wright^11^ suggest memory bias can be reduced by maximizing the time between ratings, blinding the rater from their first rating, and having a different random ordering of the data. This study aimed to minimize the amount of memory bias by following these 3 recommendations.

## Recommendations

During a disaster, responders are under a high level of stress, which may contribute to the incompleteness of patient encounter records. Nevertheless, data completeness is crucial to analysis and evaluation of responses. Prior to future deployments, the importance of data collection and completeness should be reiterated to NDMS personnel and HHS staff during IT-specific training. Diagnosing patients and entering the data into an EMR as accurately and completely as possible during an emergency response helps to inform operations and plan for future response activities of a similar nature. Most importantly, accurate and complete EMR data could support immediate needs for specific medications, supplies, and personnel during a response.^23^

One potential avenue for furthering this work is to identify if the Operational Code methodology can be applied to other disaster data sets and implemented as a real-time surveillance tool by leveraging advances in health information technology and natural language processing. It is our recommendation that the Operational Code methodology be tested in real-time to determine if this methodology can provide a more accurate assessment of the patient encounters seen within the constraints of an actual emergency response.

## Conclusion

The intent of this work was to develop and assess if providing a new combined variable (i.e., Operational Code), which reflected both primary discharge diagnosis and chief complaint data was more informative and enable better operational decision-making than either field alone. High percentages of missing data regarding patient encounters leaves a situational awareness gap, and decisions on what resource and personnel needs are emerging cannot easily be made. The methods described above for providing more details about patient encounters by using combined insight from both diagnosis and chief complaints improved knowledge about the impacts of the Haiti earthquake retrospectively. By implementing the methodology in near real-time, we could improve data utility and thereby decision-making and performance during a disaster.

## Appendix 1

### Category Names

**Table:**

ICD-9-CM Classification of Diseases and Injuries **Full Standard Names**	Shortened ICD-9-CM Classification of Diseases and Injuries Names Used
Infectious and Parasitic Diseases	Same
Neoplasms	Same
Endocrine, Nutritional, and Metabolic Diseases and Immunity Disorders	Endocrine, Nutritional, Metabolic, Immunity
Diseases of the Blood and Blood-Forming Organs	Blood and Blood-Forming Organs
Mental Disorders	Same
Diseases of the Nervous System and Sense Organs	Nervous and Sense Organs
Diseases of the Circulatory System	Circulatory
Diseases of the Respiratory System	Respiratory
Diseases of the Digestive System	Digestive
Diseases of the Genitourinary System	Genitourinary
Complications of Pregnancy, Childbirth, and the Puerperium	Pregnancy, Childbirth, Puerperium
Diseases of the Skin and Subcutaneous Tissue	Skin, Subcutaneous
Diseases of the Musculoskeletal System and Connective Tissue	Musculoskeletal and Connective Tissue
Congenital Anomalies	Same
Certain Conditions Originating in the Perinatal Period	Perinatal
Symptoms, Signs, and Ill-Defined Conditions	General
Injury and Poisoning	Same
